# HCV core antigen and HCV-RNA in HIV/HCV co-infected patients with different HCV genotypes

**DOI:** 10.1186/1471-2334-14-222

**Published:** 2014-04-23

**Authors:** Anna Rosa Garbuglia, Alessia Monachetti, Claudio Galli, Rosella Sabatini, Monica Lucia Ferreri, Maria Rosaria Capobianchi, Patrizia Bagnarelli

**Affiliations:** 1 Virology, Laboratory of Virology, “L.Spallanzani” National Institute for Infectious Diseases, Via Portuense, 292, 00149 Rome, Italy; 2 Virology Laboratory, Department of Biomedical Sciences and Public Health, Marche Polithecnic University, Via Conca 71, 60100 Ancona, Italy; 3 Scientific Affairs, Abbott Diagnostics, Via Amsterdam 125, 00144 Roma, Italy

## Abstract

**Background:**

A good correlation between HCV core antigen (HCVAg) and different HCV-RNA assays has been described, but little data are available in HCV/HIV co-infection. We aimed to evaluate HCVAg in comparison with HCV-RNA and to determine their kinetics during antiviral treatment in selected HCV/HIV co-infected patients.

**Methods:**

355 samples from 286 HCV/HIV co-infected subjects for whom HCV-RNA (Abbott RealTime) was requested were analysed also for HCVAg (Abbott ARCHITECT) in order to evaluate the correlation between the two parameters both in patients treated or untreated for chronic hepatitis C and according to different HCV genotypes. The differences between percentages were evaluated by chi square or Fisher’s exact test, while mean and median values were compared by Student’s t test or the Mann–Whitney test, respectively. All differences were considered significant for a p value <0.05.

**Results:**

HCVAg was detectable on 288/315 sera (91.4%) positive for HCV-RNA and in 5 out of40 (12.5%) sera with undetectable HCV-RNA for a total concordance of 90.1%. The correlation was fair both in untreated (r = 0.742) and in treated (r = 0.881) patients and stronger for genotypes 1 and 4 than for genotype 3. Both HCV-RNA and HCVAg levels were significantly higher (p = 0.028 and p = 0.0098, respectively) in patients infected by genotype 1 than by genotype 3. The mean ratio of Log values between HCV-RNA (IU/mL) and HCVAg (fmol/liter) was 2.27 ± 1.09 in untreated and 2.20 ± 0.82 in treated patients (p = n.s.),consistent with a sensitivity of HCVAg corresponding to about 1,000 IU/mL of HCV-RNA, and ranged from 2.21 to 2.32 among HCV genotypes with no significant differences; five samples (1.4%; 2 genotype 1a or 1c, 3 genotype 3a) showed highly divergent values. The analysis of 18 monitoring profiles from patients treated with PEG-IFN and Ribavirin showed similar trends, except in one case in which relapse could be predicted by HCVAg and not by HCV-RNA.

**Conclusion:**

These results suggest that HCVAg represents an adequate tool for determining an ongoing HCV infection also in HIV co-infected patients, with lower costs and faster turnaround time than HCV-RNA.

## Background

The hepatitis C virus (HCV) was first identified in 1989 as the principal cause of post-transfusion non-A non-B hepatitis [1]. Worldwide an estimated 170 million people are infected with HCV and, due to common routes of transmission 4–5 million are co-infected with the human immunodeficiency virus (HIV) [2,3]. The current standard for diagnosing an active HCV infection is the detection and quantification of HCV-RNA, also recommended to monitor the antiviral treatment. Therapy guidelines for standard treatment with pegylated interferon (PEG-IFN) plus Ribavirin recommend the discontinuation of the therapy if HCV-RNA decline is <2 log at week 12 or when HCV RNA is still detectable at week 24 [4]. On therapy HCV-RNA evaluation is even more crucial to tailor new therapeutic regimens based on direct acting antivirals (DAA) drugs [5]. For the measurement of HCV-RNA, a number of qualitative and quantitative assays are commercially available offering a very low limit of detection and linear quantification over a broad dynamic range [6-9]. Although the available HCV-RNA testing systems are very sensitive and have a high-throughput performance, nucleic acid tests are expensive, labour intensive, and to avoid false positive results require technical skills, which limit their use [10].

A fully automated quantitative immunoassay for measuring the HCV core antigen (ARCHITECT HCVAg, Abbott Diagnostics, Wiesbaden, Germany) was registered within EC in 2009 [11]. The assay sensitivity across the different HCV genotypes ranges from 500 to 3,000 IU/mL of HCV-RNA [12-14]. Several studies have demonstrated a good correlation between HCVAg and different HCV-RNA assays [12,13,15-17] and suggested that HCVAg could be used as an alternative to HCV-RNA testing in some settings, for instance in hemodialysis patients [18]. Moreover HCVAg has been demonstrated to considerably reduce the window period in 97% of HCV-RNA positive/anti-HCV antibody negative specimens demonstrating that it represents a reliable marker of viral replication even in HCV pre-seroconversion phase [13,19,20]. To date, little data are available about HCVAg determination in HCV/HIV co-infected patients: studies carried out so far in this population included a small number of samples and did not give robust information on potential interference of HIV replicative activity on the specificity of HCVAg determination and on its sensitivity, particularly in the subjects with low HCV RNA level (HCV-RNA <10^4^ IU/mL) [21].

The aims of the present study were to establish the clinical sensitivity and to evaluate the concordance and correlation of HCVAg with HCV-RNA in HCV/HIV co-infected patients, according to HCV genotypes, and in co-infected patients undergoing treatment for chronic hepatitis C.

## Methods

### Study population

Routine samples were selected from HCV/HIV positive patients admitted to the National Institute for Infectious Diseases (INMI) “L. Spallanzani” in Roma or to the Regional Hospital “Torrette” in Ancona for whom HCV-RNA and HIV-RNA testing had been required. The residual plasma samples were aliquoted, stored at -80°C and thawed only once for HCVAg testing. All samples come from the normal routine of our hospitals, therefore of course we did have access to the basic demographic information. However, after having collected all these data the patient list was completely anonymized, so that their ID recognition was no longer possible. In both Institutions a very large number of patients are followed for HIV and HCV infections every year, thus the age and gender information will not allow to identify them; in no way it is possible to link any study result with patient ID. The current Italian regulation on the protection of personal data have been strictly observed, in particular: “Codice in materia di protezione dei dati personali” (d.lg. 30 giugno 2003, n. 196) and “Provvedimento su amministratore di sistema”, issued by “Garante per la protezione dei dati personali” on November 27, 2008, and published in Gazzetta Ufficiale, December 24, 2008.

Samples were selected in order to obtain a balanced distribution of low, intermediate and high levels of HCV-RNA and to include sequential specimens from patients who had started PEG-IFN + RBV treatment for hepatitis C. Additionally, eleven routine specimens from HIV-positive individuals with high HIV viral load and negative for HBsAg, anti-HCV antibodies and HCV-RNA were collected in order to check the specificity of the HCVAg assay.

### Serological and virological assays

HCVAg was quantified by ARCHITECT i2000SR (Abbott Diagnostics, Wiesbaden, Germany), a fully automated system, with a chemiluminescent immunoassay already described in detail [11,12]. The linear range of HCVAg quantification spans between 3.00 and 20,000 femtomoles/liter (fmol/L) (0.06 and 400 pg/ml), with an automated 1:9 dilution that extends the assay linearity up to 180,000 fmol/L (3,600 pg/mL). Samples with results greater than the cutoff value (>3 fmol/L) but ≤10 fmol/L were retested in duplicate and considered “true” positive if confirmed reactive.

Other serological markers (anti-HCV, HIV Ag/Ab) were evaluated using the respective assays on the Abbott ARCHITECT system.

HCV-RNA was quantified by means of a commercial real-time RT-PCR assay (RealTime™ HCV, Abbott Molecular Inc, Des Plaines, Il, USA) as specified by the manufacturer. The detection limit was 12 IU/mL. HCV genotypes were determined by VERSANT HCV Genotype 2.0 Assay (LiPa) (Innogenetics, Ghent, Belgium; distributed by Siemens Healthcare Diagnostics) [22] or by Abbott RealTime HCV Genotype II Assay (Abbott) [23].

### Statistical analysis

The results were collected on a Microsoft Excel worksheet and analyzed for correlation (Spearman) and agreement (K statistic) by the Analyse-it software (Analyse Ltd, Birmingham, UK). The differences between percentages were evaluated by chi square or Fisher’s exact test, while mean and median values were compared by Student’s t test or the Mann–Whitney test, respectively. All differences were considered significant for a p value ≤0.05.

## Results

Three hundred and fifty-five samples obtained from 292 HIV/HCV co-infected patients were included in this study. Of those, 106 were obtained from 43 patients under standard treatment with PEG-IFN + Ribavirin for chronic hepatitis C and 249 were taken from as many untreated patients.The mean and median ages did not differ significantly between genders and were 46.2 ± 7.9 and 47 (range: 20–84) years overall, respectively. As outlined in the study protocol, samples were stratified according to the HCV-RNA levels (Table 1). Sequential samples during treatment were available from 41 patients (3 or more draws from 18 patients, 2 draws from 23 patients).

**Table 1:** Positivity and levels of HCVAg according to HCV-RNA levels in HIV/HCV co-infected patients

**HCV-RNA IU/mL**	**Treated**			**Untreated**			**Total**			
	**N.**	**HCVAg+**	**%**	**N.**	**HCVAg+**	**%**	**N**	**HCVAg+**	**%**	**Median**
<12	23	5	21.7	17	0	0	40	5	12.5	7.32
12-999	12	3	25.0	11	0	0	23	3	13.0	6.04
1000-9999	11	8	100	17	11	64.7	28	19	67.9	16.04
10000-99999	14	14	100	30	30	100	44	44	100	72.71
100000-999999	22	22	100	66	66	100	88	88	100	548.67
≥1000000	24	24	100	108	107	99.1	132	131	99.2	2968.20
Total	106	76	74.5	249	214	85.9	355	290	81.7	770.51

All the 11 samples from patients negative for HBV and HCV markers and anti-HIV antibody positive with HIV-RNA level >10^5^ copies/mL were negative for HCVAg.

The overall concordance between HCVAg and HCV-RNA was 90.1% and theagreement was fair (kappa = 0.61). On samples from treated patients the qualitative agreement was significantly lower than on samples from untreated patients (84.0% vs. 92.8%; p = 0.01 by chi square). On the whole then, 285 samples were positive and 35 were negative (i.e. below the detection limit) for both markers, while 30 were positive for HCV-RNA (median level: 479 IU/ml; range 13–1,598,723) and negative for HCVAg and 5, all obtained from treated patients, were positive for HCVAg (median level: 7.32 fmol/L; range: 6.71-139.96) and negative for HCV-RNA. Furthermore, 3 out of 12 with RNA levels <1,000 UI/mL were positive for HCVAg among treated patients vs. none among untreated.

The positivity rates for HCVAg according to different levels of HCV-RNA and the median levels are reported in Table 1. Based on a polynomial regression analysis between HCV-RNA and HCVAg on the 22 samples positive for both and with a viral load between 100 and 10,000 IU/mL, the positivity threshold of the HCVAg assay corresponds to about 1,000-1,500 IU/mL of HCV-RNA, and indeed 282 out of 292 samples (96.6%) containing >1,000 IU/mL of HCV-RNA were positive for HCVAg. Five specimens (1.4%) showed a highly divergent quantitative result between the two assays, either because HCVAg levels were higher than HCV-RNA levels or because the Log ratio between the two parameters exceeded the mean plus 2 standard deviations measured on all samples. Details on the discrepant specimens, either qualitative or quantitative, are reported in Table 2.

**Table 2:** Discrepant results between HCVAg and HCV-RNA

**Case**	**Genotype**	**HCVAg**	**HCV-RNA**	**Log ratio**	**Notes**
**n**		**Fmol/L**	**IU/mLI**	**RNA/Ag**	
1	3a	6.71	0	na	Therapy 12 m, log ratio at T0 1.08, neg for both at 32 m
2	1a	11.21	0	na	Therapy 8 m, log ratio at T0 2.46
2	1a	139.96	0	na	Therapy 16 m, log ratio at T0 2.46
3	3a	7.32	<12	na	Therapy 12 m, log ratio at T0 2.34, log ratio at 8 m 2.92
4	3a	6.82	<12	na	Therapy 4 m, log ratio at T0 2.43
5	na	0.00	1142	na	Untreated, single specimen
6	3a	0.20	1807	na	Untreated, single specimen
7	4	0.54	3468	na	Untreated, single specimen
8	3a	1.89	4975	na	Untreated, single specimen
9	na	1.78	7939	na	Untreated, single specimen
10	3a	1.19	9206	na	Untreated, single specimen
11	1a	2.69	1598723	14.44	Untreated, single specimen
12	1c	3,972.47	2097	0.92	Untreated, single specimen
13	1a	7.92	105551	5.59	Untreated, single specimen
14	3a	8.83	40643	4.87	Untreated, single specimen
15	3a	18.96	698555	4.57	Untreated, single specimen
16	3a	31.20	7338172	4.57	Untreated, single specimen

Five samples from four patients positive for HCVAg and negative for HCV-RNA, seven samples negative for HCVAg despite HCV-RNA levels higher than 1,000 UI/mL, five samples with a Log ratio between the two assays outside the expected range; na, not available; m, months.

**Figure 1:**
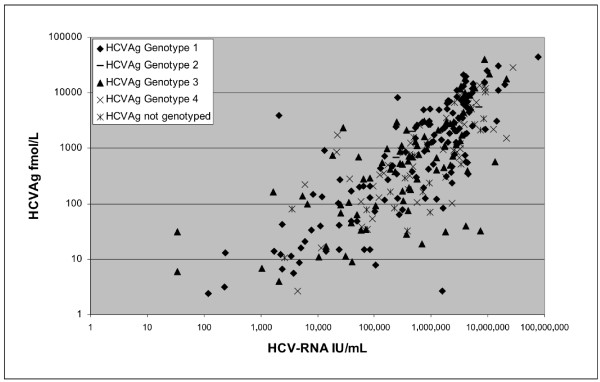
**Correlation between HCVAg and HCV-RNA on 315 samples from patients with HCV RNA >12 IU/ml.** Values are reported on a logarithmic scale and with different symbols according to genotype. The correlation coefficient (Spearman) was 0.869.

The correlation between HCV-RNA and HCVAg was good in treated patients (Spearman r = 0.881; 95% confidence limits (CL) 0.821-0.921) and fair in untreated patients (Spearman r = 0.742; 95% CL 0.675-0.797; p = 0.05). Globally, the correlation was r = 0.815 with 95% CL: 0.774-0.849 (Figure 1). The ratio between HCV-RNA and HCVAg Log levels was also evaluated: the mean ± standard deviation and median ratio values were 2.20 ± 0.82 and 1.94 in treated patients vs. 2.27 ± 1.09 and 1.99 in untreated and (p = n.s.) and 2.25 ± 0.88 and 1.98 overall.

**Figure 2:**
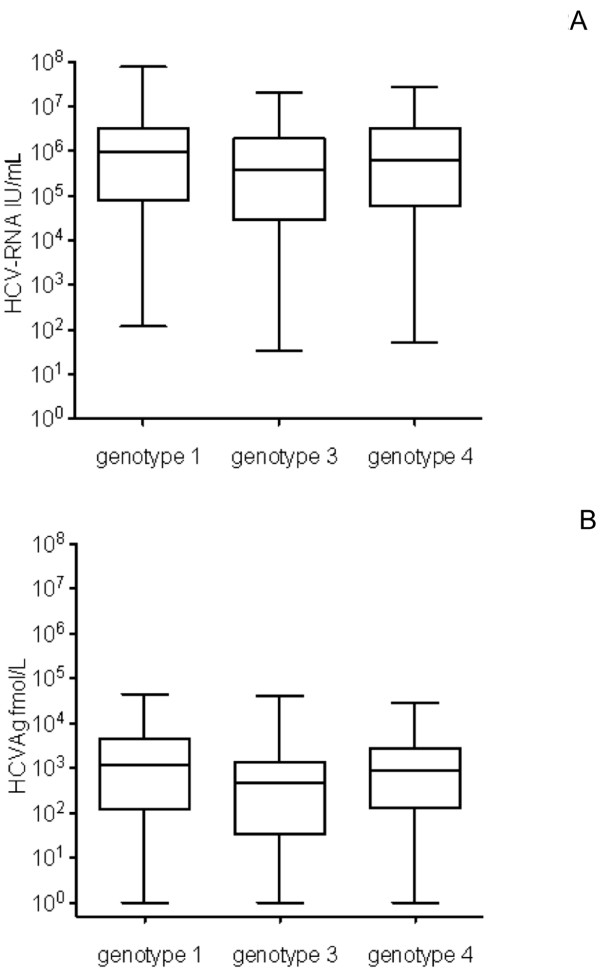
**Distribution of HCV-RNA (A) and HCVAg (B) by HCV genotype.** Significant differences between median values were observed only between genotypes 1 and 3 for both HCV-RNA (p = 0.028) and HCVAg (p = 0.0098).

The HCV genotype was available for 245 of the 286 patients (86%), while in 41 cases it was not known due to low or undetectable HCV-RNA levels. Genotypes 1, 3 and 4 were the most represented as shown in Table 3. The correlation between HCV-RNA and HCVAg was fair (Spearman r = 0.816; 95% confidence limits (CL) 0.774-0.851; p < 0.0001) (Figure 1), and was greater for genotype 1 and 4 (Spearman r = 0.82 and 0.84) than for genotype 3 (Spearman r = 0.74). The Log ratio between HCV-RNA and HCVAg was not significantly different among genotype 1 (2.22 ± 0.80 and 1.94), genotype 3 (2.32 ± 0.88 and 2.00) and genotype 4 (2.21 ± 1.05 and 1.99). The distribution of HCV-RNA and HCVAg levels according to HCV genotypes 1, 3 and 4 are reported in Figure 2. Since HCV-RNA levels and HCVAg levels were not normally distributed, the difference among genotypes was assessed by a nonparametric test (Mann–Whitney). The difference was statistically significant for both parameters in genotype 1 vs genotype 3 (p = 0.028 for HCV-RNA and p = 0.0098 for HCVAg), while the difference between genotype 4 and 3 failed to reach the statistical significance (p = 0.122 and 0.114, respectively). No difference was observed between genotype 1 and genotype 4 (p = 0.902 and 0.578, respectively).

**Table 3:** HCV genotypes in the study population of 286 HIV/HCV co-infected patients

				**Genotype 1**						**Genotype 2**			**Genotype 3**			**Genotype 4**			Mixed*
				**N (%)**						**N (%)**			**N (%)**			**N (%)**			**N(%)**
**Gender**	**Total N**	**Genotyped N (%)**	**Total**	**NS**	**a**	**b**	**c**	**a/b**	**Total**	**NS**	**a**	**a/c**	**Total**	**NS**	**a**	**Total**	**NS**	**a**	
Unknown	13	10	6	1	3	2	0	0	0	0	0	0	2	1	1	2	2	0	0
		(76.9)	(60.0)						(-)				(20.0)			(20.0)			(-)
Female	74	59	33	9	13	7	4	0	0	0	0	0	19	2	17	7	4	3	0
		(79.7)	(55.9)						(-)				(25.7)			(11.9)			(-)
Male	199	176	78	18	25	18	15	3	3	1	1	1	58	8	50	35	27	8	2
		(88.4)	(44.3)						(1.7)				(33.0)			(19.9)			(1.1)
**Total**	**286**	**245**	**117**	**28**	**41**	**27**	**19**	**2**	**3**	**1**	**1**	**1**	**79**	**11**	**68**	**44**	**33**	**11**	**2**
		**(85.7)**	**(47.8)**						**(1.2)**				**(32.2)**			**(18.0)**			**(0.8)**

N, number; NS, not subtyped; The two mixed cases (*) were infected by genotype 1a and 3 and genotype 1a and 3a. respectively. Forty-one genotypes (14.3%) were not available due to very low levels of HCV-RNA.

**Figure 3:**
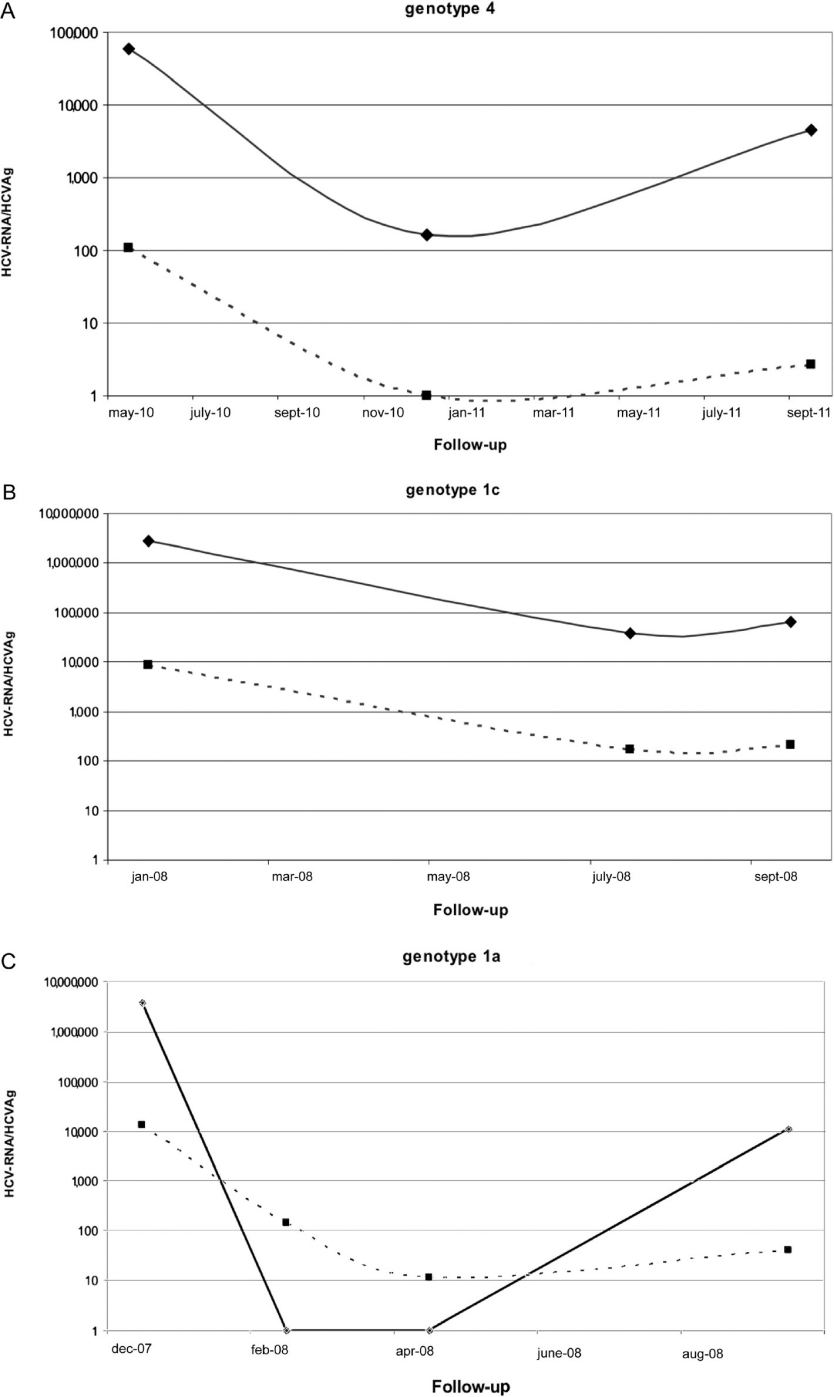
**Monitoring profiles from three patients co-infected by HCV and HIV.** The values of HCV-RNA and HCVAg are expressed on a logarithmic scale as IU/mL and fmol/L. respectively. The trends of the two parameters in the two patients described in Figure 3**A** and **B** were almost identical whereas the patient in Figure 3**C** showed a persistence of HCVAg positivity and a virological relapse on the following draw. Dotted line: HCVAg; solid line: HCV-RNA.

In 17 of the 18 patients sampled 3 or more times during PEG-IFN + Ribavirin therapy the behaviour of HCV-RNA and HCVAg was coincident, as 12 patients showed a sustained decrease, 2 patients a less pronounced decrease and 3 patients no significant variations: two examples are reported in Figure 3A and B. For one patient a significant discrepancy was observed (Figure 3C): both HCV-RNA and HCVAg declined initially, but while the former was undetectable on two consecutive draws, taken 2 months apart, HCVAg was still positive at 139.96 and 11.21 fmol/L. On the last draw, both HCV-RNA and HCVAg were detected (11,050 IU/mL and 40.20 fmol/L, respectively).

## Discussion

HCVAg has been initially proposed as a surrogate marker of HCV-RNA for blood screening [5] and soon afterwards it was suggested that the quantitative determination of this parameter could surrogate RNA testing also in the monitoring of chronic HCV infections and possibly also in patients under therapy [24-27]. The availability of a fully automated immunoassay with on-board sample pretreatment for the quantitative determination of HCVAg has brought up a series of new studies. Most of those have confirmed the original data presented by the manufacturer [11], reporting a good specificity [12,13,17] and a sensitivity in the range of 500–3,000 IU/mL of HCV-RNA, according to the HCV genotype and possibly to other factors. Indeed, the theoretical and experimental equivalency between the two parameters was investigated by Schuettler et al. [28], who established that according to the HCV structure 1 pg of HCVAg corresponds to 43,000 IU of HCV-RNA, whereas on clinical samples this ratio was much lower (1 pg = about 7,900 IU of HCV-RNA). This has been confirmed in subsequent studies based on different HCV RNA quantification systems [13-16,18]; in particular, very recently Descamps et al. [14] found a very significant correlation of HCVAg to HCV-RNA, measured by TaqMan amplification (r = 0.90; p < 0.001) and an equivalency of 1 pg of HCVAg = 9,755 IU of HCV-RNA, that equals about 600 IU/mL at the HCVAg assay threshold of 3 fmol/L. From those data, confirmed also by our observation, only a portion of the circulating HCVAg seems to be associated with complete virions, implying that a variable fraction of circulating HCVAg may be secreted by the infected cells. Additionally the HCVAg measured by the immunoassay derives also from antigen-antibody complexes that are disrupted during sample pre-treatment. Furthermore, it has been observed that also HCV, like the hepatitis B virus, may be present in the bloodstream as complete virions and defective particles, presumably with low or no infectivity [29] especially in the late phases of infection. The discrepancy between HCV-RNA and HCVAg production may be large enough in some instances to justify the huge differences observed in 1.4% of samples in our observation as well as in other studies [12,13,15] and the combined action of all the above mentioned factors may help explain why the equivalency and correlation between viral RNA and core antigen differs among the various studies.Viral variability may also be implicated in generating these discrepancies, such as the finding of one specimen with >1 million IU/mL of HCV-RNA and negative for HCVAg. For instance, it has been shown that a variability in the core gene products is able to affect the recognition of circulating HCVAg because polymorphisms at amino acids 47 to 49 of the core Ag were responsible for underestimation [30] and that the levels of HCVAg and ratio of HCV-RNA to HCVAg may be affected by the polymorphism of the IL28B genotype [31].

In our experience the correlation (r = 0.818) was weaker than reported by others, as previous studies with the same HCVAg assay reported r values up to 0.90 or even higher [14,32]. This does not seem to be ascribed to the relatively high rate (30%) of samples from treated patients in our selection, since the relationship between these two indexes of active HCV infections appears stronger in treated than in untreated patients. Recently, Kuo et al. [32] assayed 405 subjects identified by a community screening for HCVAg and found a high degree of correlation with HCV-RNA (R^2^ = 0.94), concluding that the combined use of anti-HCV levels and of HCVAg would have a very high predictive value for viremia, helping to identify individuals with active HCV infection in the screening of an asymptomatic population. Indeed, the currently available HCVAg assay is suitable for the purpose of identifying the presence of active HCV infection in anti-HCV positive individuals [16,17,33], bearing also a significant prognostic value [34].

We analyzed the potential effect of HCV-RNA levels on the RNA/Ag Log ratio separately in samples from treated and untreated patients. We did not observe any significant differences between the two groups, neither between groups with HCV-RNA levels lower or higher than 10 IU/mL of HCV-RNA.

The target population of our study was different from the majority of the other studies on HCVAg, as it was entirely composed of patients co-infected by HCV and HIV. The double infection is rather frequent, due to common routes of transmission [2,35-37], and liver disease is the second leading cause of death in patients infected by HIV [38].

In Italy HCV infection has a higher prevalence than in other Western countries [39], and IDUs have represented the highest risk group for HIV for at least 2 decades, thus the number of co-infected individuals should be quite high [39,40]. The HCV genotype distribution gives an indirect confirmation to this hypothesis: while in Italian population genotypes 1 and 2 appear the most represented [39], in our study genotype 2 was almost absent and genotype 4, mainly transmitted by parenteral routes and linked to drug injection [39,40], is rare in other settings, while it was observed in 18% of patients from the present study.

HCV replication appears to be higher in HIV co-infected than in HCV monoinfected patients as indicated by previous studies [41,42]. Thus far, only 3 very recent studies have explored the behaviour of HCV antigen in HIV/HCV co-infections. Xu et al. [43] have shown a higher variability of the HCV Core gene sequences and a more diversified HCV quasispecies population in association with the progressive decrease in the immune pressure caused by long-term HIV infection, but HIV co-infection did not seem to influence the correlation between HCV-RNA and HCVAg. In residents from a rural village in China Shen et al. [42] have found a high correlation both in 129 HCV-only infected (r = 0.808) and in 98 patients with the double infection (r = 0.952). The results of Mederacke et al. [21] were substantially similar, with a 96% concordance and a very high correlation (r = 0.97) on 71 samples from 58 co-infected individuals. Our results were different, since the concordance (91.5%) and correlation (r = 0.816) were both lower. It is also of note that ratio of HCV-RNA/HCVAg log_10_ levels reported in 16 Chinese co-infected patients [43] were lower and more homogeneous (1.78 ± 0.16; median 1.74) than in the present study (2.25 ± 0.88; median 1.98) and not different from those found in HCV-monoinfected subjects.

Several factors may help explain these discrepancies: first, our population was numerically much more consistent (355 samples from 292 patients, i.e. almost twice as many as in the 3 other studies combined). Furthermore no information on the HCV genotype was available from the latter study while in the former study [21] most samples were collected from patients infected by genotype 1 (41/71) or genotype 2 (23/71) and these Authors found a very high correlation for both, while in the present study the correlation in genotype 3 infection was weaker. Finally, a different proportion of patients undergoing treatment for HCV and host factors related to the different populations may account for the observed discrepancies observed between this and previous studies.

The kinetics of HCV-RNA and HCVAg in a subgroup of 18 co-infected patients under treatment for chronic hepatitis C was also evaluated and confirms the substantial concordance and similar temporal trends already observed in other studies [12,25,27], though in some cases the decrease of HCVAg may be slower as we found samples reactive for HCVAg and negative or with very low levels of HCV-RNA only in samples obtained during antiviral treatment for hepatitis C.We also describe one notable exception, in which the persistence of HCVAg after the disappearance of HCV-RNA from the blood stream anticipated a virological relapse. While the complete mechanisms of inhibition of viral replication and of the production of viral antigens in patients undergoing treatment for chronic hepatitis C are still unclear, testing for HCVAg during either conventional or DAA-based treatment, as recently suggested [26,27], will bring only a marginal additional cost and may enable to identify some cases that will benefit from a closer monitoring of HCV-RNA levels in order to anticipate viral rebound.

## Conclusions

Testing for HCVAg may represent a suitable option for assessing the presence and degree of active HCV infection also in patients co-infected by HIV. As in other viral infections, the adoption of standardized serological testing on high-volume automated systems will guarantee a faster turnaround time. This is particularly useful for the close monitoring requested in the new therapeutic options with direct antiviral acting drugs (DAA). In addition, this approach can help reducing the cost, especially in terms of personnel, for the initial screening and routine patient assessment and will allow to focus the time and skills on more demanding and cumbersome virological assays.

### Ethical approval

This study was exempt from ethical review, since it was based on a retrospective chart review, and analyses were performed on an anonymous database. In this respect, the local policy was employed in accordance with the recently reviewed international policy [44], and with current Italian legislation (see Methods section).

## Competing interests

Dr. Claudio Galli is currently employed by Abbott Diagnostics, Italy, as the Scientific Affairs Manager. The other Authors have no conflicts of interest to declare.

## Authors’ contributions

ARG, CG, MrC, PB conceptualised the study. Samples were collected and prepared by RS,MF,AM. The assays were carried out by RS, MF, AM, PB. ARG, CG interpreted the results, and edited the manuscript. MRC, PB participated in critical review of the paper. All authors read and approved the final version of the manuscript.

## Pre-publication history

The pre-publication history for this paper can be accessed here:

http://www.biomedcentral.com/1471-2334/14/222/prepub

## References

[B1] Choo QL Kuo G Weiner AJ Overby LR Bradley DW Houghton M Isolation of a cDNA clone derived from a blood-borne non-A, non-B viral hepatitis genome Science 1989 14 359 362 10.1126/science.2523562 2523562

[B2] Shepard CW Finelli L Alter MJ Global epidemiology of hepatitis C virus infection Lancet Infect Dis 2005 14 558 567 10.1016/S1473-3099(05)70216-4 16122679

[B3] Hatzakis A Wait S Bruix J Buti M Carballo M Cavaleri M Colombo M Delarocque-Astagneau E Dusheiko G Esmat G Esteban R Goldberg D Gore C Lok ASF Manns M Marcellin P Papatheodoridis G Peterle A Prati D Piorkowsky N Rizzetto M Roudot-Thoraval F Soriano V Thomas HC Thursz M Valla D van Damme P Veldhuijzen IK Wedemeyer H Wiessing L Zanetti AR Janssen HLA The state of hepatitis B and C in Europe: report from the hepatitis B and C summit conference J Viral Hepat 2011 14 suppl. 1 1 16 21824223 10.1111/j.1365-2893.2011.01499.x

[B4] European Association for the Study of the Liver EASL clinical practice guidelines: management of hepatitis C virus infection J Hepatol 2011 14 245 264 21371579 10.1016/j.jhep.2011.02.023

[B5] Hofmann WP Sarrazin C Zeuzem S Current standards in the treatment of chronic hepatitis C Dtsch Arztebl Int 2012 14 352 358 22675406 10.3238/arztebl.2012.0352 PMC3364529

[B6] Halfon P Bourlière M Pénaranda G Khiri H Ouzan D Real-time PCR assays for hepatitis C virus (HCV) RNA quantitation are adequate for clinical management of patients with chronic HCV infection J Clin Microbiol 2006 14 2507 2511 10.1128/JCM.00163-06 16825372 PMC1489518

[B7] Pyne MT Konnick EQ Phansalkar A Hillyard DR Evaluation of the Abbott investigational use only RealTime hepatitis C virus (HCV) assay and comparison to the Roche TaqMan HCV analyte-specific reagent assay J Clin Microbiol 2009 14 2872 2878 10.1128/JCM.02329-08 19625475 PMC2738061

[B8] Bortoletto G Campagnolo D Mirandola S Comastri G Severini L Pulvirenti FR Alberti A Comparable performance of TMA and Real-Time PCR in detecting minimal residual hepatitis C viraemia at the end of antiviral therapy J Clin Virol 2011 14 217 220 10.1016/j.jcv.2010.11.010 21195023

[B9] Bossler A Gunsolly C Pyne MT Rendo A Rachel J Mills R Miller M Sipley J Hillyard D Jenkins S Essmyer C Young S Lewinski M Rennert H AmpliPrep/COBAS TaqMan® automated system for hepatitis C virus (HCV) quantification in a multi-center comparison J Clin Virol 2011 14 100 103 10.1016/j.jcv.2010.10.020 21145783

[B10] Chevaliez S Virological tools to diagnose and monitor hepatitis C virus infection Clin Microbiol Infect 2011 14 116 121 10.1111/j.1469-0691.2010.03418.x 21054664

[B11] Morota K Fujinami R Kinukawa H Machida T Ohno K Saegusa H Takeda K A new sensitive and automated chemiluminescent microparticle immunoassay for quantitative determination of hepatitis C virus core antigen J Virol Methods 2009 14 8 14 10.1016/j.jviromet.2008.12.009 19135481

[B12] Ross RS Viazov S Salloum S Hilgard P Gerken G Roggendorf M Analytical performance characteristics and clinical utility of a novel assay for total hepatitis C virus core antigen quantification J Clin Microbiol 2010 14 1161 1168 10.1128/JCM.01640-09 20107102 PMC2849592

[B13] Medici MC Furlini G Rodella A Fuertes A Monachetti A Calderaro A Galli S Terlenghi L Olivares M Bagnarelli P Costantini A De Conto F Sainz M Galli C Manca N Landini MP Dettori G Chezzi C Hepatitis C virus core antigen: analytical performances, correlation with viremia and potential applications of a quantitative, automated immunoassay J Clin Virol 2011 14 260 265 10.1016/j.jcv.2011.05.003 21621454

[B14] Descamps V Op de Beck A Plassart C Brochot E Francois C Helle F Adler M Bourgeois N Degré D Duverlie G Castelain S Strong correlation between liver and serum levels of hepatitis C virus core antigen and RNA in chronically infected patients J Clin Microbiol 2012 14 465 468 10.1128/JCM.06503-11 22162563 PMC3264181

[B15] Mederacke I Wedemeyer H Ciesek S Steinmann E Raupach R Wursthorn K Manns MP Tillmann HL Performance and clinical utility of a novel fully automated quantitative HCV-core antigen assay J Clin Virol 2009 14 210 215 10.1016/j.jcv.2009.08.014 19766055

[B16] Kesly R Polat H Terzi Y Kurtoglu MG Uyar Y Comparison of a newly developed automated and quantitative hepatitis C virus (HCV) core antigen test with the HCV RNA assay for clinical usefulness in confirming anti-HCV results J Clin Microbiol 2011 14 4089 4093 10.1128/JCM.05292-11 21940466 PMC3233016

[B17] Kamili S Drobeniuk J Araujo AC Hayden TM Laboratory diagnostics for hepatitis C virus infection Clin Infect Dis 2012 14 S1 S43 S48 22715213 10.1093/cid/cis368

[B18] Miedouge M Saune K Kamar N Rieu M Rostaing L Izopet J Analytical evaluation of HCV core antigen and interest for HCV screening in haemodialysis patients J Clin Virol 2010 14 18 21 10.1016/j.jcv.2010.02.012 20233674

[B19] Icardi G Ansaldi F Bruzzone BM Durando P Lee S de Luigi C Crovari P Novel approach to reduce the hepatitis C virus (HCV) window period: clinical evaluation of a new enzyme-linked immunosorbent assay for HCV Core antigen J Clin Microbiol 2001 14 3110 3114 10.1128/JCM.39.9.3110-3114.2001 11526137 PMC88305

[B20] Leary TP Gutierrez RA Muerhoff AS Birkenmeyer LG Desai SM Dawson GJ A chemiluminescent, magnetic particle-based immunoassay for the detection of hepatitis C virus core antigen in human serum or plasma J Med Virol 2006 14 1436 1440 10.1002/jmv.20716 16998880

[B21] Mederacke I Potthoff A Meyer-Olson D Meier M Raupach R Manns MP Wedemeyer H Tillmann HL HCV core antigen testing in HIV- and HBV-coinfected patients, and in HCV-infected patients on hemodialysis J Clin Virol 2012 14 110 115 10.1016/j.jcv.2011.11.009 22177274

[B22] Verbeeck J Stanley MJ Shieh J Celis L Huyck E Wollants E Morimoto J Farrior A Sablon E Jankowski-Hennig M Schaper C Johnson P Van Ranst M Van Brussel M Evaluation of Versant hepatitis C virus genotype assay (LiPA) 2.0 J Clin Microbiol 2008 14 1901 1906 10.1128/JCM.02390-07 18400913 PMC2446848

[B23] Martró E González V Buckton AJ Saludes V Fernández G Matas L Planas R Ausina V Evaluation of a new assay in comparison with reverse hybridization and sequencing methods for hepatitis C virus genotyping targeting both 5′ noncoding and nonstructural 5b genomic regions J Clin Microbiol 2008 14 192 197 10.1128/JCM.01623-07 17989191 PMC2224264

[B24] Zanetti AR Romanò L Brunetto M Colombo M Bellati G Tackney C Total HCV core antigen assay: a new marker of hepatitis C viremia for monitoring the progress of therapy J Med Virol 2003 14 27 30 10.1002/jmv.10355 12629640

[B25] Takahashi M Saito H Higashimoto M Atsukawa K Ishii H Benefit of hepatitis C virus core antigen assay in prediction of therapeutic response to interferon and ribavirin combination therapy J Clin Microbiol 2005 14 186 191 10.1128/JCM.43.1.186-191.2005 15634970 PMC540104

[B26] Vermehren J Susser S Berger A Perner D Peiffer KH Allwinn R Zeuzem S Sarrazin C Clinical utility of the ARCHITECT HCV Ag assay for early treatment monitoring in patients with chronic hepatitis C genotype 1 infection J Clin Virol 2012 14 17 22 10.1016/j.jcv.2012.05.008 22698697

[B27] Loggi E Cursaro C Scuteri A Grandini E Martello Panno A Galli S Furlini G Bernardi M Galli C Andreone P Patterns of HCV-RNA and HCV Core antigen in the early monitoring of standard treatment for chronic hepatitis C J Clin Virol 2013 14 291 295 10.1016/j.jcv.2012.11.012 23245628

[B28] Schuettler CG Thomas C Discher T Friese G Lohmeyer J Schuster R Schaefer S Gerlich WH Variable ratio of hepatitis C virus RNA to viral Core antigen in patient sera J Clin Microbiol 2005 14 1977 1981 10.1128/JCM.42.5.1977-1981.2004 PMC404599 15131157

[B29] Gastaminza P Dryden KA Boyd B Wood MR Law M Yeager M Chisari FV Ultrastructural and biophysical characterization of hepatitis C virus particles produced in cell culture J Virol 2010 14 10999 11009 10.1128/JVI.00526-10 20686033 PMC2953183

[B30] Murayama A Sugiyama N Watashi K Masaki T Suzuki R Aizaki H Mizuochi T Wakita T Kato T Japanesereferencepanel of bloodspecimens for evaluation of hepatitis C virus RNA and core antigen quantitative assays J Clin Microbiol 2012 14 1943 1949 10.1128/JCM.00487-12 22495557 PMC3372108

[B31] Durante-Mangoni E Vallefuoco L Sorrentino R Lossa D Perna E Molaro R Braschi U Zampino R Sodano G Adinolfi LE Utili R Portella G Clinico-pathological significance of hepatitis C virus core antigen levels in chronic infection J Med Virol 2013 14 1913 1918 10.1002/jmv.23672 23897630

[B32] Kuo YH Chang KC Wang JH Tsai PS Hung SF Hung CH Chen CH Lu SN Is hepatitis C virus core antigen an adequate marker for community screening? J Clin Microbiol 2012 14 1989 1993 10.1128/JCM.05175-11 22461676 PMC3372126

[B33] Ergunay K Sener B Alp A Karakaya J Hascelick G Utility of a commercial quantitative hepatitis C virus core antigen assay in a diagnostic laboratory setting Diagn Microbiol Infect Dis 2011 14 486 491 10.1016/j.diagmicrobio.2011.04.011 21767705

[B34] Ohshawa M Kato K Tanno K Ikai K Fujishima Y Okayama A Turin TC Onoda T Suzuki Seropositivity for anti-HCV core antigen is independently associated with increased all-cause, cardiovascular, and liver disease-related mortality in hemodialysis patients J Epidemiol 2011 14 491 499 10.2188/jea.JE20100187 22001541 PMC3899466

[B35] Turner J Bansi L Gilson R Gazzard B Walsh J Pillay D Orkin C Phillips A Easterbrook P Johnson M Porter K Schwenk A Hill T Leen C Anderson J Fisher M Sabin C UK Collaborative HIV Cohort (UK CHIC) Study The prevalence of hepatitis C virus (HCV) infection in HIV-positive individuals in the UK – trends in HCV testing and the impact of HCV on HIV treatment outcomes J Viral Hepat 2010 14 569 577 19840365 10.1111/j.1365-2893.2009.01215.x

[B36] Taylor LE Swan T Mayer KH HIV coinfection with hepatitis C virus: evolving epidemiology and treatment paradigms Clin Infect Dis 2012 14 S1 s33 s42 22715212 10.1093/cid/cis367 PMC3491862

[B37] Anggorowati N Yano Y Heriyanto DS Hinonce HT Utsumi T Mulya DP Subronto YW Hayashi Y Clinical and virological characteristics of hepatitis B or C virus co-infection with HIV in Indonesian patients J Med Virol 2012 14 857 865 10.1002/jmv.23293 22499006

[B38] Righi E Beltrame A Bassetti M Lindstrom V Mazzarello G Dentone C Di Biagio A Ratto S Viscoli C Therapeutical aspects and outcome of HIV/HCV coinfectedpatients treated with pegylated interferon plus ribavirin in an Italian cohort Infection 2008 14 358 361 10.1007/s15010-008-7319-5 18642111

[B39] Cornberg M Razavi HA Alberti A Bernasconi E Buti M Cooper C Dalgard O Dillion JF Flisiak R Forns X Frankova S Goldis A Goulis I Halota W Hunyady B Lagging M Largen A Makara M Manolakopoulos S Marcellin P Marinho RT Pol S Poynard T Puoti M Sagalova O Sibbel S Simon K Wallace C Young K Yurdaydin C A systematic review of hepatitis C virus epidemiology in Europe, Canada and Israel Liver Internat 2011 14 suppl. 2 30 60 10.1111/j.1478-3231.2011.02539.x 21651702

[B40] Stroffolini T D’Egidio PF Aceti A Filippini P Puoti M Leonardi C Almasio PL and the DAVIS (Drug Addicted, HCV Prevalence in Italy) participating Centers An epidemiological, observational, cross-sectional, multicenter study: Hepatitis C virus infection among drug addicts in Italy J Med Virol 2012 14 1608 1612 10.1002/jmv.23370 22930509

[B41] Danta M Semmo N Fabris P Brown D Pybus OG Sabin CA Bhagani S Emery VC Dusheiko GM Klenerman P Impact of HIV on host-virus interactions during early hepatitis C virus infection J Infect Dis 2008 14 1558 1566 10.1086/587843 18419344

[B42] Shen T Chen X Zhang W Xi Y Cao G Zhi Y Wang S Xu C Wei L Lu F Zhuang H A higher correlation of HCV core antigen with CD4+ T cell counts compared with HCV RNA in HCV/HIV-1 coinfected patients PLoS ONE 2011 14 e23550 10.1371/journal.pone.0023550 21858166 PMC3155566

[B43] Xu C-H Shen T Zheng J-J Tu J Zhang W-D Lu F-M Higher dN/dS ratios in the HCV core gene, but not in the E1/HVR1 gene, are associated with human immunodeficiency virus-associated immunosuppression Arch Virol 2012 14 2153 2162 10.1007/s00705-012-1390-z 22825695

[B44] Millum J Menikoff J Streamlining ethical review Ann Intern Med 2010 14 153 655 657 21079221 10.7326/0003-4819-153-10-201011160-00008 PMC4714763

